# Characteristics and reasons non-urgent self-referred patients visit the emergency department in Kimberley, South Africa

**DOI:** 10.4102/hsag.v30i0.3015

**Published:** 2025-09-15

**Authors:** Abimbola A. Fagbiye, Chika K. Egenasi, Wilhelm J. Steinberg, Mathew O. Benedict, Talat Habib, Francois C. van Rooyen

**Affiliations:** 1 Department of Family Medicine, Faculty of Health Sciences, University of the Free State, Bloemfontein, South Africa; 2 Department of Family Medicine, Robert Mangaliso Sobukwe Hospital, Kimberley, South Africa; 3 Department of Biostatistics, Faculty of Health Sciences, University of the Free State, Bloemfontein, South Africa

**Keywords:** self-referral, reasons, green triaged, emergency department, Kimberley, South Africa

## Abstract

**Background:**

In many countries, emergency departments (EDs) are overcrowded by self-referred, non-urgent patients, leading to strained resources. Understanding why patients with minor ailments choose secondary or tertiary EDs is vital.

**Aim:**

This study aimed to describe patients’ characteristics and reasons for self-referrals for non-urgent conditions to the ED of a tertiary hospital in Kimberley, South Africa.

**Setting:**

The study was conducted at a tertiary hospital in Kimberley.

**Methods:**

This descriptive cross-sectional study involved patients at Kimberley’s Family Medicine ED (Gateway Centre). Participants completed a self-administered questionnaire with staff available to explain.

**Results:**

A total of 331 participants were interviewed; the mean age was 40 years (ranging from 18–89 years); most were males (199; 60.1%), ≤ 45 years (226; 68.3%), single (171; 51.7%), unemployed (181; 54.7%), had no medical insurance (306; 92.5%) and had no access to a private doctor (298; 90.0%). Most had access to primary healthcare (PHC) clinics (291; 87.9%). They perceived their medical conditions as severe enough to visit the casualty department (310; 93.7%). They were more familiar with the services at the ED than their local clinics (169; 75.5%).

**Conclusion:**

Non-urgent ED visits by self-referred patients result from complex medical, psychosocial, and economic factors. Despite access to PHC, patients often prefer the ED due to perceived severity and familiarity with hospital services.

**Contribution:**

Patients’ perceptions significantly influence their healthcare choices. Addressing these perceptions is key to reducing the burden on already strained emergency services.

## Introduction

The National Department of Health’s Referral Policy for South African Health Services and Referral Implementation Guidelines recommend that patients access health services at the primary level of care closest to their homes (National DoH 2020). Primary healthcare (PHC) clinics aim to effectively deliver healthcare, ensuring accessibility, affordability, efficiency, and fair distribution of services to their respective communities. Many patients, however, choose to receive care at an emergency department (ED) for minor ailments that could be managed at the primary care level. This practice is viewed as an ‘inappropriate use’ of hospital-based EDs (Alnasser et al. 2023; Al-Otmy 2020; Keizer Beache & Guell 2016). Research shows that this practice contributes to overcrowding and overuse of resources in EDs (Alnasser et al. 2023; Gulacti & Lok 2018; Matifary et al. 2021), negatively affecting the quality of service delivery and causing patient dissatisfaction (Keizer Beache & Guell 2016). The use of emergency services for non-urgent conditions is a global concern, and many healthcare workers find such visits inappropriate regardless of the context in which they occur (Matifary et al. 2021).

The reasons behind the trend in ED attendance for non-urgent conditions are multifaceted and complex. Access, financial reasons, perceived severity of the health conditions, convenience and proximity to the ED, the expectation of special investigations, and the lack of confidence in primary care practitioners were cited as reasons for attending EDs rather than PHC clinics (Fatima et al. 2021; Minderhout et al. 2019). Patients’ expectations of better healthcare were cited as reasons for visiting EDs rather than clinics (Matifary et al. 2021). Because many patients have trauma-related complaints, some feel they can only be treated at hospitals offering X-rays (Al-Otmy et al. 2020; Fatima et al. 2021; Henninger, Spencer & Pasche 2019; Koce, Randhawa & Ochieng 2019).

Minderhout et al. (2019) in the Netherlands found that self-referred patients preferred hospitals because of perceived convenience and better available facilities. The lack of information regarding where to go and poor access to their general practitioners (GPs) also played a role (Minderhout et al. 2019). Fatima et al. (2021) reported similar findings in a study investigating the factors influencing general practice-type presentations to the ED in a rural Australian community. A qualitative study from Switzerland found that the quality of their relationship with the GP influences patients’ decisions on whether to consult the GP or visit an ED (Henninger et al. 2019).

Al-Otmy et al. (2020) reported that 78.5% of patients visiting the ED of a tertiary hospital in western Saudi Arabia did so for non-urgent conditions. The primary reasons for self-referral were the patients’ perception that their condition was urgent and the easy accessibility of the ED. Similarly, a Nigerian study found that 60% – 90% of patients self-refer to higher levels of care due to limited understanding of the healthcare system, perceptions of symptom severity, beliefs about better equipment and facilities in the ED, and advice from family or friends (Koce et al. 2019).

Self-referrals to inappropriate levels of care were associated with a lack of access to PHC in South Africa and the ease with which non-urgent patients could access hospitals. Other factors contributing to their health-seeking behaviour were their perception of their illness as serious, the lack of medical insurance, and the time of day they sought treatment (Keizer Beache & Guell 2016; Pillay & Mahomed 2019; Rajman & Mahomed 2019).

A systematic review by Uscher-Pines et al. (2013) estimated that around 30% of ED visits in the United States are for non-urgent conditions. Factors such as younger age, the greater convenience of the ED compared to other care options, and negative perceptions of non-ED care significantly contribute to these high rates. The authors suggest that the large volume of non-urgent ED visits reflects limited access to primary care and warn against the common assumption that simply redirecting non-urgent patients to other settings is an effective policy solution. They also highlight that efforts to discourage non-urgent ED use may lead to unintended consequences.

Behrens and Morgan (2023) developed an evidence-based framework to explain why people choose to visit the ED instead of a PHC facility. They identified demographic factors, such as age and deprivation, as important contributors. Additionally, the framework highlights the role of community resource organisations and patients’ perceptions, which often reflect underlying unmet needs. The authors emphasise that planning interventions should be approached with empathy and compassion towards patients.

Yeh et al. (2022), in a study conducted in Northern Taiwan, reported that while ED visits generally declined during the coronavirus disease 2019 (COVID-19) epidemic, patient numbers rebounded once the pandemic subsided.

In many parts of the world, triage scores classify patients according to their need for urgent medical care (Pillay & Mahomed 2019). A triage scale was developed in Cape Town in 2004, known as the South African Triage Scale (SATS). Many South African healthcare facilities have adopted this method, including the Robert Mangaliso Sobukwe Hospital (RMSH), a tertiary hospital in Kimberley, in the Northern Cape Province of South Africa (Pillay & Mahomed 2019; Uscher-Pines et al. 2013).

As a part of the SATS, the Triage Early Warning Scores (TEWS) assign a triage colour code based on the score; TEWS assign a 0–2 score as green (non-urgent), 3–4 score as yellow (urgent), 5–6 score as orange (very urgent) and ≥ 7 scores as red (emergency). The SATS assigns a blue code to deceased patients (Pillay & Mahomed 2019). Triage nurses at RMSH assign these triage colours primarily based on the measured vital signs and any additional clinical condition (discriminator) that warrants urgent attention (for example, chest pain) (Pillay & Mahomed 2019). A patient’s triage colour code determines their priority level (Pillay & Mahomed 2019). For this research, the SATS was used as a coding system to identify the ‘green’ non-urgent patients. The system only recognises medical or clinical factors when classifying patients as non-urgent. Patients often present to the ED without referral letters due to underlying social, economic or psychological causes (Behrens et al. 2023), but the assessment of these factors is not included in the triage forms.

Overcrowding and long waiting times in the ED at RMSH, Kimberley, have raised concerns. Patient dissatisfaction stemming from extended waiting periods has been documented through formal complaint channels (personal communication with the quality assurance department). The overcrowding issue, partly driven by self-referred patients seeking care for non-urgent conditions, leads to the excessive utilisation of resources originally designated for urgent and emergency cases within the ED.

This study aimed to describe adult patients’ characteristics and reasons for self-referral to RMSH Emergency Department, Kimberley, who were green-triaged (non-urgent) based on SATS criteria from May to July 2021.

## Research methods and design

### Study design

This study was a descriptive cross-sectional study of patients presenting to Kimberley’s Family Medicine Emergency Department (Gateway Centre) from 01 May to 31 July 2021 (3 months).

### Study setting

Robert Mangaliso Sobukwe Hospital (Kimberley Hospital) is in the city of Kimberley, Northern Cape province. It is a regional and tertiary hospital affiliated with Universitas Academic Hospital of the University of the Free State. The hospital has two EDs, namely, ‘Gateway Centre’, which is the family medicine ED run by family physicians, and ‘Emergency Centre’, which is run by emergency physicians. The Emergency Centre receives patients categorised by triage as orange and red cases, while the Gateway Centre receives yellow and green patients. Yellow and green patients seen in the Gateway Centre are managed, discharged or referred to other specialities as required.

The Gateway Centre is open 24 h a day, 7 days a week, including public holidays. On weekdays, it operates three shifts: 08:00–16:00, 16:00–22:00, and 22:00–08:00. On weekends and public holidays, there are two 12-h shifts: 08:00–20:00 and 20:00–08:00.

Throughout regular working hours, a consultant family physician is present with at least four doctors available in the Gateway Centre: Registrar, Senior Medical Officer, Community Service Medical Officer and Intern. After hours, the cadre of doctors mentioned above is still present, except for the family physician, who is on standby and can be called in for hands-on assistance as and when necessary. During each shift, there is a single triage unit with two triage-trained nurse practitioners assigned to triage every patient presenting to the ED and distribute the patients to the relevant ED based on the assigned colour code.

According to Robert Mangaliso Sobukwe Hospital ED’s monthly statistics around the study period, approximately 2000 patients were directed to the Gateway Centre following triage. Among them, 75% – 80% were adults, and 43% – 48% were triaged as green. Additionally, 85% – 90% of the patients were self-referred.

Kimberley has 1 community health centre (CHC) and 13 PHC clinics open from Monday to Friday from 08:00 to 16:00, and they see both walk-in and booked patients. General practice offices are open between 08:00 and 17:00 from Monday to Friday and between 08:00 and 14:00 during weekends. Emergency cases are referred to RMSH via the ED. There is no district hospital in Kimberley, and no after-hour medical services are available in the public sector outside RMSH. The study was conducted during office hours on weekdays, as patients had no alternative in the public sector apart from the ED if they needed medical care after hours.

### Study population

The study population consists of self-referred adult patients presenting to the ED and triaged as green during regular office hours between 08:00 and 16:00, from Mondays to Fridays.

### Definition of self-referral

Any person who presents at the hospital or higher level of care for examination, medication or treatment without a referral.

### Definition of green triage

Any person who is assigned a ‘green’ triage category (non-urgent) based on the South African Triage Scale (SATS).

### Inclusion criteria

Patients aged 18 years and above who presented to the Gateway Centre between 08:00 and 16:00 from Monday to Friday, triaged as green without a referral letter and consented to participate in the study were recruited.

### Exclusion criteria

Patients with referral letters triaged as green and patients presenting after 16:00 on weekdays, weekends and public holidays were excluded. Patients whose triage status was upgraded due to worsening medical conditions were also excluded.

### Sampling method

This study used a consecutive sampling method to obtain data. Patients who presented in Gateway Centre and met the inclusion criteria were recruited if they consented to participate in the study.

### Sample size

The average monthly attendance was about 2000 patients during the study period, as mentioned earlier. The sample size was recalculated at 323 with the Raosoft sample size calculator at a 95% confidence interval and an error margin of 5%. The study recruited 331 participants. All 331 questionnaires were complete and none were discarded.

### Measurements

A confidential, structured questionnaire with 22 questions was administered to the participants. The first author designed the questionnaire using information obtained from reading relevant literature. Participants completed a self-administered questionnaire with staff available to explain.

The questionnaire is divided into two sections. Section one includes eight demographic questions covering age, sex, marital status, employment status, use of chronic medications, and medical insurance. Section two contains 14 yes-or-no questions regarding the reasons for visiting the ED, followed by one open-ended question where participants are asked to provide an explanation.

Two questions were about the accessibility of the local clinics, and another two were about the availability of regular family doctors and the convenience of ED visits. A further four questions looked at the availability of services at the clinic. Another question asked if the participants felt their condition was serious enough for an ED visit and if they came to see the doctor for a second opinion. The last question asked the participants to give other reasons for their visits and to explain. The questionnaires were completed while still in triage. After completing the survey, the questionnaire was returned to the first author or his assistant, and the participant could proceed to see the doctors in the Gateway ED. The questionnaire was available in English, Afrikaans and Setswana – the major spoken languages in the Northern Cape. Completion of the questionnaire did *not cause treatment delay*.

### Pilot study

The study was piloted in the Gateway Centre with 10 participants who met the inclusion criteria. This helped in testing the questionnaire and the project processes. No changes were made to the questionnaire, and the data were incorporated into the final analysis.

## Statistical analysis

Descriptive statistics, namely means and standard deviations or medians and percentiles, were calculated for continuous data. Frequencies and percentages were calculated for categorical data. The analysis was done by the Department of Biostatistics at the University of the Free State, Bloemfontein. The data analyses were performed using SAS software, version 9.4, from the SAS Institute Inc., Cary, NC, United States.

### Ethical considerations

Approval to conduct this research was obtained from the Health Sciences Research Ethics Committee (HSREC) of the University of Free State, with ethics approval number UFS-HSD2020/1158/2411. Permission was also obtained from the Northern Cape Department of Health with reference number NC_2020RMSH_011 to conduct the study in Kimberley.

Number coding was used to ensure the confidentiality of the participant’s responses. No names or personal identifiers appeared on any research-related information or datasheet sent for statistical analysis. The researcher kept all paper-based records in a secure location, only accessible to those involved in the study. All information was managed in a confidential manner.

## Results

A total of 331 participants were recruited for the study; the mean age ± s.d. was 40 ± 16.36 years (ranging from 18 to 89 years). Table 1 shows the other demographic characteristics of the participants. Over half of the participants were unemployed (*n* = 181, 54.7%). Only 25 (7.6%) participants had medical insurance.

**TABLE 1:** Demographic characteristics of participants (*N* = 331).

Demographic characteristics	Frequency (*n*)	%
**Gender**		
Male	199	60.1
Female	131	39.6
Other	1	0.3
**Age (years)**		
18–29	117	35.4
30–45	109	32.9
46–59	54	16.3
60+	51	15.4
**Relationship status**		
Single	171	51.7
Living together	35	10.6
Married	93	28.1
Separated	8	2.4
Divorced	24	7.3
**Employment status**		
Employed or self-employed	150	45.3
Unemployed	181	54.7
**Social grant recipients**		
Yes	93	28.1
No	238	71.9
**Had chronic medical condition(s)**		
Yes	124	37.5
No	207	62.5
**Taking chronic medication(s)† (*n* = 124)**		
Yes	106	85.5
No	18	24.5
**Had medical insurance**		
Yes	25	7.6
No	306	92.5
**Had access to PHC clinic**		
Yes	291	87.9
No	40	12.1
**Also attend the local PHC clinic for medical needs**		
Yes	224	67.7
No	107	32.3

*Source*: Adapted from Fagbiye, A.A., Habib, T., Steinberg, W.J. & Van Rooyen, F.C., 2023, *Patients’ characteristics and reasons for self-referral to emergency department at Robert Mangaliso Sobukwe Hospital Kimberley with non-urgent conditions*, e-poster, South African Academy of Family Physicians, viewed 14 July 2025, from https://saafp.org/wp-content/uploads/2023/08/Final-ePoster.pdf

PHC, primary healthcare.

† , *n* = 124 (participants having chronic medical conditions).

Most participants (*n* = 291, 87.9%) reported having access to PHC clinics. Of the 40 (12.1%) that reported a lack of access to the PHC clinics, it was due to reasons such as ‘distance from the clinic’ (25%), ‘no clinic’ (52.5%), ‘insufficient medication in the clinic’ (7.5%), ‘no doctor in the clinic’ (2.5%), ‘delay in getting help’ (5%) and ‘I live closer to the hospital’ (5%).

Most participants (*n* = 298, 90.1%) had no access to a private doctor. Among the participants who attended a local clinic in the past for various medical needs (*n* = 224), about three-quarters (*n* = 169, 75.5%) indicated that they were more familiar with the services rendered in the RMSH ED than in their PHC clinics, thus preferring to attend RMSH ED to obtain X-rays, CT scans, and other laboratory tests, etc.

Two hundred and thirty-five participants (71.0%) presented to the Gateway ED because they believed their medical problems required special investigations that could only be done at the hospital. Most participants (*n* = 310, 93.7%) believed that the severity of their medical conditions warranted attending the Gateway ED rather than their PHC clinics. This information is depicted in Table 2. Other reasons were grouped according to the frequency of related responses, as shown in Table 3.

**TABLE 2:** Reasons for Gateway Emergency Department attendance by the participants (*N* = 331).

Reasons	Frequency (*n*)	%
**No access to a family doctor or general practitioner**		
Yes	298	90.0
No	33	10.0
**Gateway emergency was closer to the participant’s residence than the local PHC clinic or GP**		
Yes	65	19.6
No	266	80.4
**The participants attended a local clinic but were more familiar with resources at RMSH than the local PHC clinics (*n* = 224)†**		
Yes	169	75.5
No	53	23.7
Not applicable	2	0.9
**Chronic medication ran out of stock at the local PHC clinic (*n* = 106)** ‡		
Yes	13	12.3
No	93	87.7
**Need for chronic script renewal (*n* = 106)**‡		
Yes	35	33.0
No	71	67.0
**Need for special investigations**		
Yes	235	71.0
No	96	29.0
**Had social problems**		
Yes	44	13.3
No	287	86.7
**Perception of having a serious medical condition**		
Yes	310	93.7
No	21	06.4
**Need for a second opinion**		
Yes	56	16.9
No	275	83.1

*Source*: Adapted from Fagbiye, A.A., Habib, T., Steinberg, W.J. & Van Rooyen, F.C., 2023, *Patients’ characteristics and reasons for self-referral to emergency department at Robert Mangaliso Sobukwe Hospital Kimberley with non-urgent conditions*, e-poster, South African Academy of Family Physicians, viewed 14 July 2025, from https://saafp.org/wp-content/uploads/2023/08/Final-ePoster.pdf

RMSH, Robert Mangaliso Sobukwe Hospital; PHC, primary healthcare; GP, general practitioners.

† , *n* = Total number of participants who attended a local clinic for medical needs in the past.

‡ , *n* = Total number of participants on chronic medication.

**TABLE 3:** Other reported reasons for self-referral to Gateway Emergency Department.

Other reasons	Frequency (*n*)	%
Musculoskeletal pains	60	18.1
COVID-19-related issues	50	15.1
Trauma or physical assault	30	9.4
Gastrointestinal symptoms	26	7.9
Urogenital problems	22	6.6
Surgical issues	16	4.8
Check-up or follow-up	11	3.3
Paperwork†	10	3.0
Headaches	9	2.7
Domestic (intimate partner) violence	6	1.8
Animal bites	5	1.5
Dizziness	5	1.5
Ear, nose and throat	4	1.2
Atypical chest pain‡	3	0.9
Malaise or unwell	3	0.9
Dental	3	0.9
Syncope or seizures	3	0.9
Psychiatry	2	0.6
Ophthalmology	1	0.3

COVID-19, coronavirus disease 2019.

† , J88 forms, social grant forms, sick notes, insurance claim forms, medical report forms following injuries on duty.

‡ , Neither related to cardiovascular chest pains nor potential acute coronary syndromes.

## Discussion

This study examined the characteristics and reasons why green-triaged patients present to the RMSH Family Medicine Gateway Centre. Patients’ self-referral greatly affects waiting time and incurs more costs for the healthcare systems (Rajman & Mahomed 2019). Many green-triaged self-referred participants were males, single, unemployed and not on a social grant. Similar findings were reported by Rajman and Mahomed (2019) in a study of self-referred patients at a hospital in KwaZulu-Natal, where the majority of participants were male, single and unemployed. They also reported that most of their patients were less than 39 years of age, which is in keeping with the current study, where a significant majority were between 18 and 45 years (Rajman & Mahomed 2019). The reason for this trend may be due to the younger, male, unemployed patients seeking employment in the areas near the hospital and preferring to access the hospital that is nearer to them rather than having to travel back home to their clinics.

The majority of participants in this study reported having no chronic medical conditions, which is similar to the findings by Krause et al. in patients with spinal injuries with self-reported ED visits (Krause, Cao & DiPiro 2022). Another study by Idil et al. (2018) in Turkey had similar findings, with 80.3% of self-referred patients presenting with no chronic disease. In the current study, this is understandable, considering the younger age bracket of the majority of the participants.

Most participants had no medical insurance and access to a GP, probably because they were unemployed and could not afford the cost of a general practice consultation. This is supported by the findings of Maseko and Harris (2018), who explored public perceptions of private and public hospitals in their study. It explored the financial aspects of private and public hospitals, including affordability, public trust and the acceptability of healthcare services. Kraaijvanger et al. (2016), in a meta-analysis of the motives for self-referral to the ED, described affordability as one of the main reasons for self-referral.

Insufficient resources at different healthcare facilities to offer diagnostic services like blood tests or radiological examinations to patients who require them, along with the need to establish an adequate number of clinics in every community, maintaining proper medication supplies, and ensuring regular doctor visits to clinics for patient support may lead individuals to circumvent existing referral procedures and seek assistance in facilities where they can obtain these services. This can result in an excessive burden on these facilities. This study found that some of the reasons given for bypassing the PHC clinics and local GPs to self-refer to the ED were similar to findings from other studies such as ‘needs for special investigations’, ‘radiologic or blood investigations to get a diagnosis’, ‘perceptions of having serious medical conditions’, ‘no clinic’, ‘insufficient medication in the clinic’, ‘no doctor in the clinic’ and ‘delay in getting help at the clinic’ (Abere, Atnafu & Mulu 2021; Henninger et al. 2019; Kraaijvanger et al., 2015, 2016; Pillay & Mahomed 2019).

Most of the self-referred participants in this study presented commonly with musculoskeletal pain, COVID-19-related symptoms and physical injuries. While numerous studies conducted during the COVID-19 pandemic reported a decline in overall ED attendance (Fyntanidou et al. 2022; Lateef 2020), Daniels et al. (2021) observed a contrasting trend, and he reported an increase in ED visits among frequent attenders presenting with medically unexplained symptoms (Daniels et al. 2021). Musculoskeletal pain and injuries were reported by Kraaijvanger et al. (2015) as being the common reasons for ED visits by self-referred patients in a Dutch hospital. Participants in the current study may have self-referred because they perceived that their medical condition was severe enough to warrant radiological imaging or more potent analgesics than what is available in other healthcare facilities.

### Recommendations

It is recommended that healthcare authorities in the Northern Cape should develop emergency caseload management policies to improve the efficiency of EDs in the Northern Cape.

In the triage, suitable referral systems should be established and implemented to re-direct green self-referred patients to the proper healthcare facilities. As an alternative, PHC nurse practitioners could be hired to attend to green self-referred patients during regular and after-hours, allowing the ED to concentrate on providing care to patients needing urgent attention.

The government should ensure that PHC facilities are well-equipped and adequately staffed. Access to these facilities can also be enhanced by subsidising patient transport to and from the PHC facilities.

Primary healthcare facilities in South Africa are nurse-driven; appropriate continuing education on the proper approach to common chronic conditions should be encouraged for PHC providers. Regular audits should be carried out to ensure compliance with guidelines on managing these chronic conditions.

Educational programmes that will help inform patients about the need to adhere to the referral system should be implemented; this will help reduce overcrowding in the EDs and free up healthcare providers to focus on attending to acute emergencies. Any intervention should be patient-centred and designed with empathy and compassion to lessen the burden of EDs.

### Limitations of the study

This research took place at the height of the COVID-19 pandemic in South Africa, which impacted the volume of patients seeking care at the Gateway Emergency Centre.

The study employed a consecutive sampling approach for data collection, which might have introduced sampling bias into the study’s results. Additionally, the data were exclusively gathered from a single ED, limiting the generalisability of the study’s findings to a broader population.

The research relied on the SATS as a basis for assigning triage colour codes to patients, possibly leading to an overestimation or underestimation of the number of patients categorised as having a green triage colour.

## Conclusion

This study looked at the characteristics and reasons of self-referred, green-triaged patients presenting to the RMSH ED in Kimberley. These patients were mostly male, single, less than 45 years and unemployed who believed they had serious conditions requiring urgent medical care and specialised investigations. It is necessary for the provincial health department to educate patients about the services available at the PHC clinics and the appropriate use of EDs.

## References

[CIT0001] Abere, T.M., Atnafu, D.D. & Mulu, Y., 2021, ‘Self-referral and associated factors among patients attending adult outpatient departments in Debre tabor general hospital, North West Ethiopia’, *BMC Health Services Research* 21(1), 1–8. 10.1186/s12913-021-06642-7 34183005 PMC8240286

[CIT0002] Alnasser, S., Alharbi, M., AAlibrahim, A., Aal ibrahim, A., Kentab, O., Alassaf, W. et al., 2023, ‘Analysis of emergency department use by non-urgent patients and their visit characteristics at an academic center’, *International Journal of General Medicine* 16, 221–232. 10.2147/IJGM.S391126 36711428 PMC9880025

[CIT0003] Al-Otmy, S.S., Abduljabbar, A.Z., Al-Raddadi, R.M. & Farahat, F., 2020, ‘Factors associated with non-urgent visits to the emergency department in a tertiary care centre, western Saudi Arabia: Cross-sectional study’, *BMJ Open* 10(10), e035951. 10.1136/bmjopen-2019-035951 PMC7539577 33028545

[CIT0004] Behrens, D.A., Morgan, J.S., Krczal, E., Harper, P.R. & Gartner, D., 2023, ‘Still looking in the wrong place: Literature-based evidence of why patients really attend an emergency department’, *Socio-Economic Planning Sciences* 90, 101707. 10.1016/j.seps.2023.101707

[CIT0005] Daniels, N.F., Ridwan, R., Barnard, E.B., Amanullah, T.M. & Hayhurst, C., 2021, ‘A comparison of emergency department presentations for medically unexplained symptoms in frequent attenders during COVID-19’, *Clinical Medicine* 21(4), e399–e402. 10.7861/clinmed.2020-1093 34016583 PMC8313219

[CIT0006] Fagbiye, A.A., Habib, T., Steinberg, W.J. & Van Rooyen, F.C., 2023, *Patients’ characteristics and reasons for self-referral to emergency department at Robert Mangaliso Sobukwe Hospital Kimberley with non-urgent conditions*, e-poster, South African Academy of Family Physicians, viewed 14 July 2025, from https://saafp.org/wp-content/uploads/2023/08/Final-ePoster.pdf.

[CIT0007] Fatima, Y., Hays, R., Knight, S., Neilson, A., Fleming, R., Panaretto, K. et al., 2021, ‘Drivers of general practice–type presentations to the emergency department in a remote outback community’, *Australian Journal of Rural Health* 29(3), 391–398. 10.1111/ajr.12706 34051017

[CIT0008] Fyntanidou, B., Stavrou, G., Apostolopoulou, A., Gkarmiri, S. & Kotzampassi, K., 2022, ‘Emergencies in the COVID-19 era: Less attendances, more admissions’, Cureus 14(6), e25971. 10.7759/cureus.25971 35855234 PMC9286014

[CIT0009] Gulacti, U. & Lok, U., 2018, ‘Non-urgent adult patients in the emergency department’, *Turkish Journal of Emergency Medicine* 18(3), 123. 10.1016/j.tjem.2018.06.002 30191192 PMC6107970

[CIT0010] Henninger, S., Spencer, B. & Pasche, O., 2019, ‘Deciding whether to consult the GP or an emergency department: A qualitative study of patient reasoning in Switzerland’, *European Journal of General Practice* 25(3), 136–142. 10.1080/13814788.2019.1634688 31272245 PMC6713128

[CIT0011] Idil, H., Kilic, T.Y., Toker, İ., Dura Turan, K. & Yesilaras, M., 2018, ‘Non-urgent adult patients in the emergency department: Causes and patient characteristics’, *Turkish Journal of Emergency Medicine* 18(2), 71–74. 10.1016/j.tjem.2017.10.002 29922734 PMC6005911

[CIT0012] Keizer Beache, S. & Guell, C., 2016, ‘Non-urgent accident and emergency department use as a socially shared custom: A qualitative study’, *Emergency Medicine Journal* 33(1), 47–51. 10.1136/emermed-2014-204039 25841166 PMC4717374

[CIT0013] Koce, F., Randhawa, G. & Ochieng, B., 2019, ‘Understanding healthcare self-referral in Nigeria from the service users’ perspective: A qualitative study of Niger state’, *BMC Health Services Research* 19, 1–14. 10.1186/s12913-019-4046-9 30940134 PMC6444603

[CIT0014] Kraaijvanger, N., Leeuwen, H., Van, Rijpsma, D. & Edwards, M., 2016, ‘Motives for self-referral to the emergency department: A systematic review of the literature’, *BMC Health Services Research* 16, 1–19. 10.1186/s12913-016-1935-z 27938366 PMC5148909

[CIT0015] Kraaijvanger, N., Rijpsma, D., Leeuwen, H.V. & Edwards, M., 2015, ‘Self-referrals in the emergency department: Reasons why patients attend the emergency department without consulting a general practitioner first – A questionnaire study’, *International Journal of Emergency Medicine* 8, 1–6. 10.1186/s12245-015-0096-x 26644131 PMC4671987

[CIT0016] Krause, J.S., Cao, Y. & DiPiro, N.D., 2022, ‘The relationship of secondary and chronic health conditions with emergency department visits and related hospitalizations among people with traumatic spinal cord injury’, *Archives of Physical Medicine and Rehabilitation* 103(12), 2338–2344. 10.1016/j.apmr.2022.05.004 35644216

[CIT0017] Lateef, F., 2020, ‘The impact of the COVID-19 pandemic on emergency department attendance: What seems to be keeping the patients away?’, *Journal of Emergencies, Trauma, and Shock* 13(4), 246–251. 10.4103/JETS.JETS_133_20 33897139 PMC8047960

[CIT0018] Maseko, L. & Harris, B., 2018, ‘People-centeredness in health system reform. Public perceptions of private and public hospitals in South Africa’, *South African Journal of Occupational Therapy* 48(1), 22–27. 10.17159/2310-3833/2018/vol48n1a5

[CIT0019] Matifary, C.R., Wachira, B., Nyanja, N. & Kathomi, C., 2021, ‘Reasons for patients with non-urgent conditions attending the emergency department in Kenya: A qualitative study’, *African Journal of Emergency Medicine* 11(1), 113–117. 10.1016/j.afjem.2020.09.004 33680731 PMC7910189

[CIT0020] Minderhout, R.N., Venema, P., Vos, H.M.M., Kant, J., Bruijnzeels, M.A. & Numans, M.E., 2019, ‘Understanding people who self-referred in an emergency department with primary care problems during office hours: A qualitative interview study at a daytime general practice cooperative in two hospitals in The Hague, The Netherlands’, *BMJ Open* 9(6), e029853. 10.1136/bmjopen-2019-029853 PMC6588995 31175200

[CIT0021] National Department of Health (NDoH), 2020, *Referral policy for South African health services and referral implementation guidelines*, South Africa: National Department of Health, Tshwane, viewed 22 July 2025, from https://knowledgehub.health.gov.za/elibrary/referral-policy-south-african-health-services-and-referral-implementation-guidelines.

[CIT0022] Pillay, I. & Mahomed, O.H., 2019, ‘Prevalence and determinants of self referrals to a District-Regional Hospital in KwaZulu Natal, South Africa: A cross sectional study’, *Pan African Medical Journal* 33, 4. 10.11604/pamj.2019.33.4.16963 PMC6607454 31303949

[CIT0023] Rajman, A. & Mahomed, O.H., 2019, ‘Prevalence and determinants of self-directed referrals amongst patients at hospitals in eThekwini District, KwaZulu-Natal 2015’, *South African Family Practice* 61(2), 53–59. 10.1080/20786190.2019.1582213

[CIT0024] Uscher-Pines, L., Pines, J., Kellermann, A., Gillen, E. & Mehrotra, A., 2013, ‘Deciding to visit the emergency department for non-urgent conditions: A systematic review of the literature’, *American Journal of Managed Care* 19(1), 47. 23379744 PMC4156292

[CIT0025] Yeh, C.C., Chien, C.Y., Lee, T.Y. & Liu, C.H., 2022, ‘Effect of the COVID-19 pandemic on emergency department visits of patients with an emergent or urgent diagnosis’, *International Journal of General Medicine* 15, 4657–4664. 10.2147/IJGM.S362615 35548587 PMC9081622

